# Progress in Polyurethane and Composites

**DOI:** 10.3390/polym16142031

**Published:** 2024-07-17

**Authors:** Chang-An Xu, Zhuohong Yang

**Affiliations:** 1 Institute of Chemical Engineering, Guangdong Academy of Sciences, Guangzhou 510665, China; 2 Key Laboratory for Biobased Materials and Energy of Ministry of Education, College of Materials and Energy, South China Agricultural University, Guangzhou 510642, China; yangzhuohong@scau.edu.cn

## 1. Introduction

Polyurethane materials have received increasing attention as daily materials due to their unique structures and properties. Polyurethane polymers are a class of polymers containing repeated carbamate groups in their main chain, which are formed according to the polyaddition of isocyanates and polyols [1]. By changing the type and composition of the raw materials, the shape and properties of polyurethane products can be adjusted. Due to this adaptivity, along with their excellent wear resistance, impact resistance, biocompatibility, adhesion, and mechanical properties, they are widely used as adhesives, elastomers, foam, and medical materials. However, pure polyurethane materials are limited by their structure, with their performance unable to meet the application requirements in some specific environments. Therefore, this is encouraging researchers to continue to explore and innovate polyurethane materials through the modification of the polyurethane structure or by creating composites with other materials to improve its performance and expand its potential applications.

With the increasing depletion of fossil fuel resources, enhanced environmental awareness, and China’s proposal of its “dual carbon” goals, sustainable and green polyurethane materials are becoming increasingly popular. Waterborne polyurethane is a type of polyurethane material based on water as the dispersing medium, making it a green, environmentally protective, and pollution-free product expected to replace traditional oily polyurethane. Waterborne polyurethane reduces the volatilization of organic solvents and reduces harm to the human body. Equally, as its use is in line with green chemistry and sustainable development strategies, its study is sure to become a future direction of research. However, waterborne polyurethane is not as efficient as solvent-based polyurethane in terms of its resistance to water and high temperatures. With expansion of the research, these shortcomings of waterborne polyurethane are expected to be resolved.

In view of analyzing polyurethane materials and their development trends, 10 research papers and 1 review article on the synthesis and modification of polyurethane materials were published in this Special Issue “Progress in Polyurethane and Composites”. Herein, the properties of polyurethane were characterized using various research methods and discussed, which expanded the possibilities for its application in different fields and provided a valuable point of reference for polyurethane-related research. These articles are introduced as follows.

## 2. An Overview of the Published Articles

In the study by Serrano-Martinez et al., in order to alleviate the crisis of non-renewable fossil fuel usage and reduce the harm of traditional polyurethane adhesives to the environment [2], the authors used a high-temperature treatment to extract lignin from rice straw as a raw polyol material and synthesize a polyurethane adhesive, effectively substituting traditional polyol PPG with lignin. The performance of this environmentally friendly adhesive and its application in the footwear industry were evaluated through thermogravimetric analysis, rheological analysis, and T-peel testing. The results showed that when the lignin content was 7.5%, the thermal and mechanical properties of the adhesive were effectively maintained, and the adhesive showed excellent thermal stability. This work increased the added value of lignin and expanded its application in the field of adhesives, in line with green chemistry and sustainable development strategies.

In Lee et al.’s study, a polyurethane acrylate prepolymer (UA) was prepared using polytetrahydrofuran, polyethylene glycol, and polypropylene glycol as polyols and acrylic monomer as the end-sealing agent. This was then polymerized with polymethyl methacrylate (PMMA) to synthesize a room-temperature binder with high strength and high transparency [3]. When PMMA was blended with UA, the physical entanglement between the two polymers formed a three-dimensional network structure, which improved the mechanical properties of the adhesive. By using molecular design to change the type and content of polyols used, the polymer adhesives were prevented from yellowing during curing reactions. The results showed that when the UA value was 5~10%, the light transmittance, shear strength, and tensile strength of the prepared polymer adhesive were high, and its properties were customizable. The PMMA/UA binder prepared in this work is expected to be a promising candidate for future road marking polymer binders.

Because of their unique molecular structure and renewable properties, vegetable oils have gradually become a research focus. Using castor oil, maleic anhydride, and glyceryl methacrylate as their raw materials, Tuo et al. successfully prepared a new multifunctional castor-oil-based acrylate (MACOG) through two-step chemical modification. They then prepared a castor-oil-based waterborne polyurethane acrylate emulsion and finally prepared a series of coating materials under UV curing [4]. The results showed that with an increase in the MACOG content, the glass transition temperature increased from 20.3 °C to 46.6 °C, and the surface water contact angle increased from 73.85 °C to 90.57 °C. In addition, the thermal decomposition temperature, mechanical strength, and water resistance of the sample were also greatly improved, mainly on account of the introduction of MACOG, which improved the system’s cross-linking density. This study not only provided new ideas for the preparation of waterborne polyurethane coatings with excellent comprehensive properties but also expanded the application of castor oil in coatings.

Enhancing the mechanical properties of polymer composites by using fiber materials to enhance the interfacial interaction has been deemed an efficient method. In the study by Ünal et al., non-functional graphene nanosheets (GNPs) were combined with a water-based, highly branched, multifunctional polyurethane dispersion (HBPUD) to effectively regulate the fiber–matrix interface in FRPCs [5]. By means of a unique ultrasonic spray deposition technique, the GNP/HBPUD aqueous-phase mixture was deposited on the surface of carbon fiber fabric to prepare epoxy prepreg sheets and corresponding FRPC laminates. The influence of the polyurethane (PU) and GNP contents and their ratio at the fiber–matrix interface on the tensile properties of the resulting high-performance composites was systematically investigated using stress–strain analysis of the produced FRPC plates and SEM analysis of their fractured surfaces. Synergistic stiffening and toughening effects were observed when as low as 20 to 30 mg of the GNPs was deposited per square meter on each side of the carbon fiber fabric in the presence of the multi-functional PU layer. This resulted in a significant improvement in the tensile strength from 908 to 1022 MPa while maintaining the initial Young’s modulus or slightly improving it from approximately 63 to 66 MPa. This study underscored the importance of carefully tuning the GNP content and the PU:GNP ratio to tailor the tensile properties of high-performance CFRPCs.

In Chen et al.’s study, vinyl-capped cationic waterborne polyurethane (CWPU) was prepared using isophorone diisocyanate (IPDI), polycarbonate diol (PCDL), trimethylolpropane (TMP), and N-methyldiethanolamine (MDEA) as the raw materials and hydroxyethyl methacrylate (HEMA) as a capping agent [6]. Then, a crosslinked FPUA composite emulsion was prepared according to core–shell emulsion polymerization, with polyurethane (PU) as the shell, fluorinated acrylate (PA) as the core, and CWPU as the seed emulsion, together with dodecafluoroheptyl methacrylate (DFMA), diacetone acrylamide (DAAM), and methyl methacrylate (MMA). The effects of the core–shell ratio of PA/PU on the surface properties, mechanical properties, and heat resistance of the FPUA emulsions and films were investigated. The results showed that when w(PA) = 30~50%, the highest-stability FPUA emulsion was generated, and under TEM, the particles displayed a core–shell structure with bright and dark intersections. When w(PA) = 30%, the tensile strength reached 23.35 ± 0.08 MPa. When w(PA) = 50%, the fluorine content on the surface of the coating film was 14.75%, and the contact angle was as high as 98.5°, indicative of good hydrophobicity. AFM was used to observe the surface flatness of the films. It was found that the tensile strength of the films increased and then decreased with an increase in the core–shell ratio, and the heat resistance of the FPUA films gradually increased. The FPUA film had excellent properties, such as good impact resistance, high flexibility, high adhesion, and corrosion resistance.

Enhancing the dielectric strength and minimizing the dielectric loss of insulation materials have piqued the interest of many researchers as enhancement techniques. It is worth noting that the electrical breakdown of insulation material is determined by its electrochemical and mechanical performance. Optimizing the mechanical, electrical, and chemical properties of new materials is considered during the generation process. Thermoplastic polyurethane (TPU) is often used as a high-voltage insulator due to its favorable mechanical properties, high insulation resistance, lightweight nature, recovery, large actuation strain, and cost-effectiveness. Its elastomer structure enables its application in a broad range of high-voltage (HV) insulation systems. In the study by Ersoy et al., the feasibility of using TPU as a solid insulator instead of a pressure plate for transformer windings was evaluated [7]. Their experimental investigation shed light on the potential of TPU to expand the range of insulating materials used in HV transformers. Transformers play a crucial role in HV systems; hence, the selection of suitable materials, like cellulose and polyurethane, is of the utmost importance. This study involved the preparation of an experimental laboratory setup. Breakdown tests were conducted by generating a non-uniform electric field using a needle–plane electrode configuration in a test chamber filled with mineral oil. Various voltages ranging from 14.4 kV to 25.2 kV were applied to induce electric field stress with a step rise of 3.6 kV. The partial discharges and peak numbers were measured based on predetermined threshold values. This study investigated and compared the behaviors of two solid insulating materials under differing non-electric field stress conditions. Harmonic component analysis was utilized to observe the differences between the two materials. Notably, at 21.6 kV and 25.2 kV, polyurethane demonstrated superior performance compared to the pressboard with regards to the threshold value for the leakage current.

Metal corrosion poses a substantial economic challenge in a technologically advanced world. In the study by Al-otaibi., novel, environmentally friendly, anticorrosive graphene oxide (GO)-doped organic–inorganic hybrid polyurethane (LFAOIH@GO-PU) nanocomposite coatings were developed using Leucaena leucocephala oil (LLO) [8]. The formulation was produced through the amidation of LLO to form diol fatty amide, followed by the reaction of tetraethoxysilane and a dispersion of GO_x_ (X = 0.25, 0.50, and 0.75 wt%), along with the reaction of isophorane diisocyanate (25–40 wt%) to form LFAOIH@GO_x_-PU_35_ nanocomposites. A detailed examination of the LFAOIH@GO_0.5_-PU_35_ morphology was conducted using X-ray diffraction, scanning electron microscopy, energy-dispersive X-ray spectroscopy, and transmission electron microscopy. These studies revealed their distinctive surface roughness features, along with a contact angle of around 88 G.U, preserving their structural integrity at temperatures of up to 235 °C with minimal loading of GO. Additionally, with the dispersion of GO, their mechanical properties were improved, including their scratch hardness (3 kg), pencil hardness (5H), impact resistance, bending, gloss value (79), crosshatch adhesion, and thickness. Electrochemical corrosion studies involving Nyquist, Bode, and Tafel plots provided clear evidence of the coatings’ outstanding anticorrosion performance.

Polyurethane (PU) composites are increasingly used as repair materials for civil engineering infrastructure, including runways, road pavements, and buildings. Evaluation of polyurethane grouting (PUG) material is critical for its maintenance. In Haruna et al.’s study, the flexural behavior of normal concrete repaired with polyurethane grout (NC-PUG) was evaluated under three-point bending tests [9]. A finite element (FE) model was developed to simulate the flexural response of the NC-PUG specimens. The equivalent principle response of the NC-PUG was analyzed through a three-dimensional finite element model (3D FEM). The NC and PUG’s properties were simulated using the stress–strain relationships determined in compressive and tensile tests. The overlaid PUG material was prepared by mixing PU and quartz sand and overlayed on either the top or bottom surface of a concrete beam. Two different overlaid thicknesses were applied, namely 5 mm and 10 mm. The composite NC-PUG specimens were formed by casting a PUG material using different overlaid thicknesses and configurations. The reference specimen showed the highest average ultimate flexural stress of 5.56 MPa ± 2.57% at a 95% confidence interval, with a corresponding midspan deflection of 0.49 mm ± 13.60%. However, due to the strengthening effect of the PUG layer, the deflection of the composite specimen was significantly improved. The concrete specimens with PUG retrofitted at the top surface demonstrated a typical linear pattern from the initial loading stage to complete failure. Moreover, the concrete specimens with PUG retrofitted at the bottom surface exhibited two deformation regions before complete failure. The FE analysis showed good agreement between the modeled numerical and experimental test results. The numerical model accurately predicted the flexural strength of the NC-PUG beam, slightly underestimating Ke by 4% and overestimating the ultimate flexural stress by 3%.

To improve the film-forming ability of hard-type acrylic latex, waterborne polyurethane–acrylate (WPUA) was grafted with polyurethane. To balance its film-forming ability and hardness, the WPUA latex was designed with a hard core (polyacrylate) and a soft shell (polyurethane) [10]. The grafting ratio was controlled by varying the content of 2-hydroxyethyl methacrylate (HEMA) used to cap the ends of the polyurethane prepolymer. The morphologies of the latex particles, the film surface, and the fracture surface of the film were characterized through transmission electron microscopy, atomic force microscopy, and scanning electron microscopy, respectively. An increase in the grafting ratio resulted in enhanced miscibility of the polyurethane and polyacrylate but reduced the adhesion between particles and increased the minimum temperature for film formation. In addition, grafting was essential to obtain transparent WPUA films. Excessive grafting induced defects such as micropores within the film, leading to decreased hardness and adhesive strength. The optimal HEMA content for the preparation of a WPUA coating with an excellent film-forming ability and high hardness in ambient conditions was 50%. The final WPUA film was prepared without coalescence agents that generate volatile organic compounds.

Aqueous polyurethane is an environmentally friendly, low-cost, high-performance resin with good abrasion resistance and strong adhesion. Cationic aqueous polyurethane has limited use in cathodic electrophoretic coatings due to its complicated preparation process and its poor stability and performance after emulsification and dispersion. The introduction of perfluoropolyether alcohol (PFPE-OH) and the application of light curing technology can effectively improve the stability of aqueous polyurethane emulsions and thus enhance the functionality of coating films. In Chen et al.’s study, a new UV-curable fluorinated polyurethane-based cathodic electrophoretic coating was prepared by using cationic polyurethane as a precursor, introducing PFPE-OH capping, and grafting hydroxyethyl methacrylate (HEMA) [11]. The results showed that the presence of perfluoropolyether alcohol in the structure affected the variation in the moisture content of the paint film after flash evaporation. Based on the emulsion particle size and morphology tests, the fluorinated cationic polyurethane emulsion was identified as a core–shell structure with its hydrophobic ends encapsulated in the polymer and its hydrophilic ends on the outer surface. After abrasion testing and baking, the fluorine atoms of the coating were found to increase from 8.89% to 27.34%. The static contact angle of the coating to water was 104.6° ± 3°, and water droplets rolled off it without traces, indicating that the coating was hydrophobic. The coating had excellent thermal stability and tensile properties and proved effective when its impact resistance, flexibility, adhesion, and resistance to chemical corrosion in extreme environments were tested. This study provided novel insights into the construction of a new and efficient cathodic electrophoretic coating system, as well as a greater scope for the promotion of cationic polyurethane in practical applications.

The inability of wounds to heal effectively through normal repair has become a burden that seriously affects socio-economic development and human health. Therapy for acute and chronic skin wounds still poses great clinical difficulty due to the lack of suitable functional wound dressings. While dressings made of polyurethane exhibit excellent diverse biological properties, they are not functional for clinical needs, and most dressings are unable to dynamically adapt to microenvironmental changes during the healing process of chronic wounds at different stages. Therefore, the development of multifunctional polyurethane composite materials has become a hot research topic. In light of this, the final review describes how the incorporation of different polymers and fillers into polyurethane dressings changes their physicochemical and biological properties and describes their applications in wound repair and regeneration. Liang et al. [12] cover several polymers, mainly natural-based polymers (e.g., collagen, chitosan, and hyaluronic acid) and synthetic-based polymers (e.g., polyethylene glycol, polyvinyl alcohol, and polyacrylamide), and some other active ingredients (e.g., LL37 peptide, platelet lysate, and exosomes). The design, conversion, use, and application of advanced functional polyurethane-related dressings are discussed, alongside future development directions, providing reference for novel designs and applications.

## 3. Conclusions

This Special Issue covers research on the structural design and performance characterization of polyurethane materials and their composites, as well as their applications in adhesives, coatings, and UV resins. Effective design and characterization are essential in creating performant polyurethane materials with wide applications. With continuous research on polyurethane materials, new multifunctional and high-performance polyurethane materials will continue to emerge. In addition, given national and international interest in green and environmentally friendly materials, the preparation of sustainable water-based materials and bio-based polyurethane materials will be the future development trend. In short, the research on polyurethane materials collated in this Special Issue constitutes a valuable point of reference for researchers working with related polyurethane materials.
